# Diagnostic and Management Perspectives in Alveolar Echinococcosis: Review of Literature

**DOI:** 10.5152/eurasianjmed.2022.22308

**Published:** 2022-12-01

**Authors:** Fahri Aydin, Ahmet Yalcin, Adem Karaman, Recep Sade, Gurkan Ozturk, Fatih Alper

**Affiliations:** 1 Ataturk University, Faculty of Medicine, Department of Radiology, Erzurum, Turkey; 2 Ataturk University, Faculty of Medicine, Department of General Surgery, Erzurum, Turkey

**Keywords:** Alveolar echinococcosis, radiologic imaging, distant organ metastases

## Abstract

Alveolar echinococcosis is a life-threatening zoonotic disease caused by *Echinococcus multilocularis*. The disease usually primarily involves the liver and shows tumor-like growth. Early diagnosis of alveolar echinococcosis is difficult because the disease is usually asymptomatic in the early stages. Untreated cases are fatal and result in death within 10 years of liver involvement.

In the diagnosis of alveolar echinococcosis, the patient's medical history, radiological imaging findings, and serological and histopathological tests are used together. Radiological imaging methods are very important for early diagnosis and differential diagnosis of the disease.

In this article, we wanted to review the diagnosis and treatment of alveolar echinococcosis and emphasize the importance of keeping it in mind, especially in cystic lesions of the liver, and the importance of early diagnosis of the disease.

**Main Points**

- Alveolar echinococcosis is a life-threatening disease with tumor-like growth.
- The combined use of radiological imaging methods is very important in early diagnosis. Clinical history and laboratory findings contribute significantly to the diagnosis.
- Depending on the type of alveolar echinococcosis lesions, they may have different imaging features. Tumoral lesions of the liver should be kept in mind in the differential diagnosis.
- It should be kept in mind that alveolar echinococcal lesions may contain micro- or macrocystic components, may be completely solid, and may contain calcifications.

## Introduction

Alveolar echinococcosis is a chronic parasitic disease caused by *Echinococcus multilocularis* (*E. multilocularis*). Human is an incidental intermediate host in the life cycle of *E. multilocularis*.^1,2^ Transmission to humans occurs by direct contact or by drinking contaminated water, resulting in tapeworm eggs being transported to the digestive tract. In particular, the liver is the first site of involvement, where metacestodes form tumor-like growths and cause chronic infection. Early diagnosis is difficult because the disease is usually asymptomatic at an early stage. Therefore, when diagnosed, alveolar echinococcosis may have caused local invasion and destruction in the liver and adjacent organs, as well as metastases to the lung, brain, bone, and other distant organs. Although organ metastases are rare, the disease is fatal if left untreated and results in death within 10 years after liver involvement.

Radiologic imaging methods are very valuable for diagnosing the disease of alveolar echinococcosis because of detecting primary organ involvement and distant organ spread. Ultrasound, which is also frequently used in the follow-up of the disease, is the first imaging method used in the diagnosis of alveolar echinococcosis. Computed tomography and magnetic resonance imaging (MRI) are extremely important for the characterization of alveolar echinococcosis lesions, the presence of local invasion, and the detection of distant organ metastases. Imaging methods are also of great importance in terms of guiding the treatment of the disease and deciding on treatment options.

## Epidemiology and Life Cycle of *Echinococcus multilocularis*

Alveolar echinococcosis is an endemic disease, especially in Central Europe, Russia, Central Asian republics, and some countries of the Middle East.^3,4^ In Turkey and especially in the region we live in (Eastern Turkey), alveolar echinococcosis is an important life-threatening zoonotic disease.^5,6^ In addition, due to the high human mobility recently, the disease has become an important health problem for other parts of Europe and Asia.

Wild canids such as foxes, wild dogs, wolves, and jackals form the definitive host group in the life cycle of *E. multilocularis*.^7,8^ While rodents, deer, and bison are intermediate hosts.^9,10^ *Echinococcus multilocularis* eggs released into the environment by definitive hosts are ingested by intermediate hosts.^11,12^ In these hosts, metacestodes begin to develop. Metacestodes contain structures called daughter cysts. These daughter cysts contain hundreds of protoscolices capable of forming adult tapeworms.

In this life cycle, human is the accidental intermediate host. After ingestion of contaminated food and drink, the eggs are distributed in the digestive tract and the embryos reach the liver via the lymphatic or portal venous system. The growth pattern of *E. multilocularis* larvae shows a tumor-like pattern in the liver and may cause destroy surrounding tissues, and invasion of vessels and the biliary tract.^13,14^

### Diagnosis and Management

Alveolar echinococcosis may have a long asymptomatic period in the early period.^15,16^ This incubation period can last between 5 and 15 years. Therefore, the diagnosis is usually incidental in the early stages of the disease. During this period, the disease is usually diagnosed in patients who have undergone radiological imaging to investigate another disease.^17^ As the liver lesion grows and destroys the surrounding tissues, liver dysfunctions and biliary system disorders may occur.^18^ Consequently, fatal complications such as chronic liver failure may occur in the advanced stage. In addition, the disease can cause death by metastasizing to vital organs such as the brain and lungs.

The patient's medical history, radiological imaging findings, and serological and histopathological tests are used together in the diagnosis of this disease.^19,20^

Echinococcus species-specific serum antibodies are detected with high diagnostic sensitivity and specificity in blood tests. The Em16 and Em18 antigens are *E. multilocularis*-specific antigens that are commonly used for diagnosis. These antigens are also used in the long-term follow-up of the disease after medical treatment. Although serological tests and radiological imaging studies are helpful, a definitive diagnosis of the disease is based on histopathological examination.

### The Role of Imaging Methods

Radiologic imaging methods are important in the detection of primary and metastatic lesions in the disease of alveolar echinococcosis. The primary alveolar echinococcosis lesion is most commonly located in the right lobe of the liver. Primary involvement of other organs such as the lung or brain has been reported as extremely rare.^21^ Few cases of alveolar echinococcosis with primary brain involvement have been reported.^22,23^

**Figure 1. A,B.**
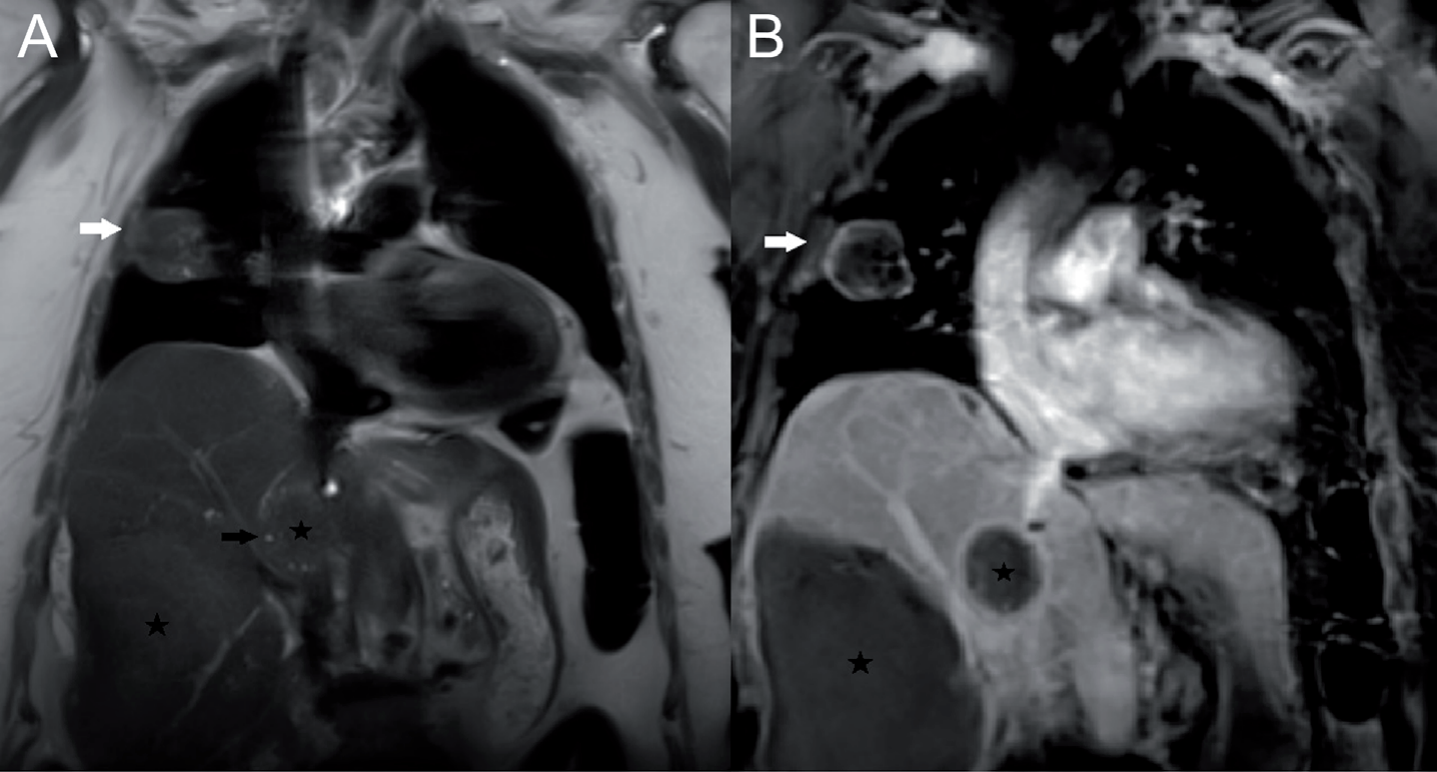
Coronal T2-weighted magnetic resonance image (A) and coronal T1-weighted contrast-enhanced image (B) show alveolar echinococcosis lesions with microcysts and solid components in the liver (black stars). The black arrow indicates the microcystic content in the lesions observed in the liver. The patient also had another alveolar echinococcosis lesion invading the pleura in the right lung (white arrow).

In alveolar echinococcosis lesions, cystic and solid components may coexist in varying proportions. In addition, calcific changes are frequently encountered within the lesions. According to the Kodama classification based on radiological imaging features, 5 types of hepatic alveolar echinococcosis cysts have been defined.^24-26^ Type 1 lesions refer to lesions that consist of multiple small cysts and do not contain solid components. Type 2 lesions contain multiple small cysts with a solid component (Figure 1). Type 3 lesions have a solid component associated with an irregular large cystic lesion. Type 4 lesions consist of solid tissue without cystic components. Type 5 lesions have only a large cystic component. Alveolar echinococcosis lesions are mostly seen as Type 2 and Type 3 lesions. Type 2 and type 3 lesions occur in approximately 40% and 46%, respectively.^27,28^ The presence of solid components in alveolar echinococcosis lesions may suggest malignant pathologies such as cystadenocarcinoma or cholangiocarcinoma in the differential diagnosis. Therefore, the presence of contrast enhancement in radiological imaging is valuable for the differentiation of malignancies from alveolar echinococcosis. Type 5 lesions consisting of only the cystic component are included in the differential diagnosis with simple hepatic cysts of the liver, and type 4 lesions consisting of only solid components are included in the differential diagnosis with solid tumors of the liver. With the contribution of advanced radiological imaging methods such as diffusion-weighted MRI and computed tomography perfusion, which are increasingly used in recent years, the diagnosis of lesions among the differential diagnoses can be made to a large extent.^29-31^ However, with the contribution of serological and histopathological evaluations, the diagnosis of alveolar echinococcosis can be confirmed.

Ultrasonography is usually the initial method in detecting liver lesions.^32,33^ Typical features detected in ultrasonography are a large mass with irregular borders, cystic areas that may represent necrosis, echogenic foci of calcifications, and internal hypo- and hyperechoic areas.^34^ Sometimes small clustered hyperechoic areas can be observed within the lesions. Ultrasonography is also valuable in demonstrating complications of the biliary system. With Doppler ultrasonography, the vascular structures in the regions adjacent to the lesion can be evaluated and information about the invasion, displacement, and thrombus formation can be obtained, if it occurs.

**Figure 2. A,B.**
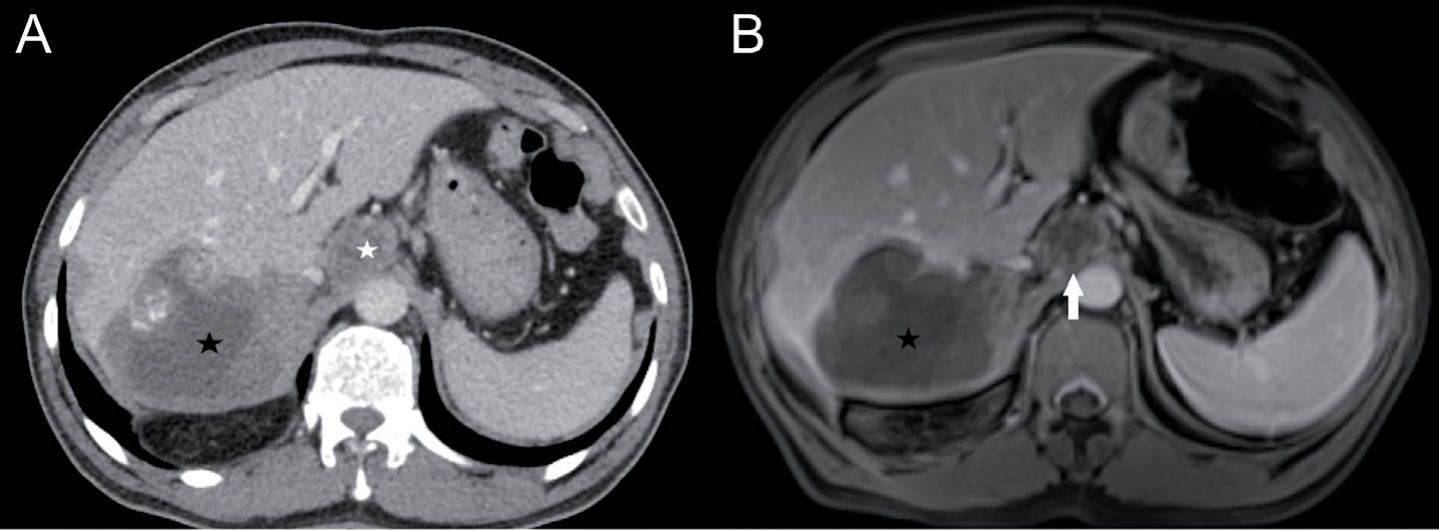
Axial contrast-enhanced computed tomography (CT) (A) and T1-weighted contrast-enhanced image (B) show alveolar echinococcosis lesions located in the liver (black stars) and anterior to the aorta (white star). Note that the liver lesion is of heterogeneous density and contains areas of hyperdense that may represent calcification in CT image (A). On magnetic resonance imaging (B), the lesion observed adjacent to the aorta is seen to invade the diaphragm (white arrow).

**Figure 3. A-D.**
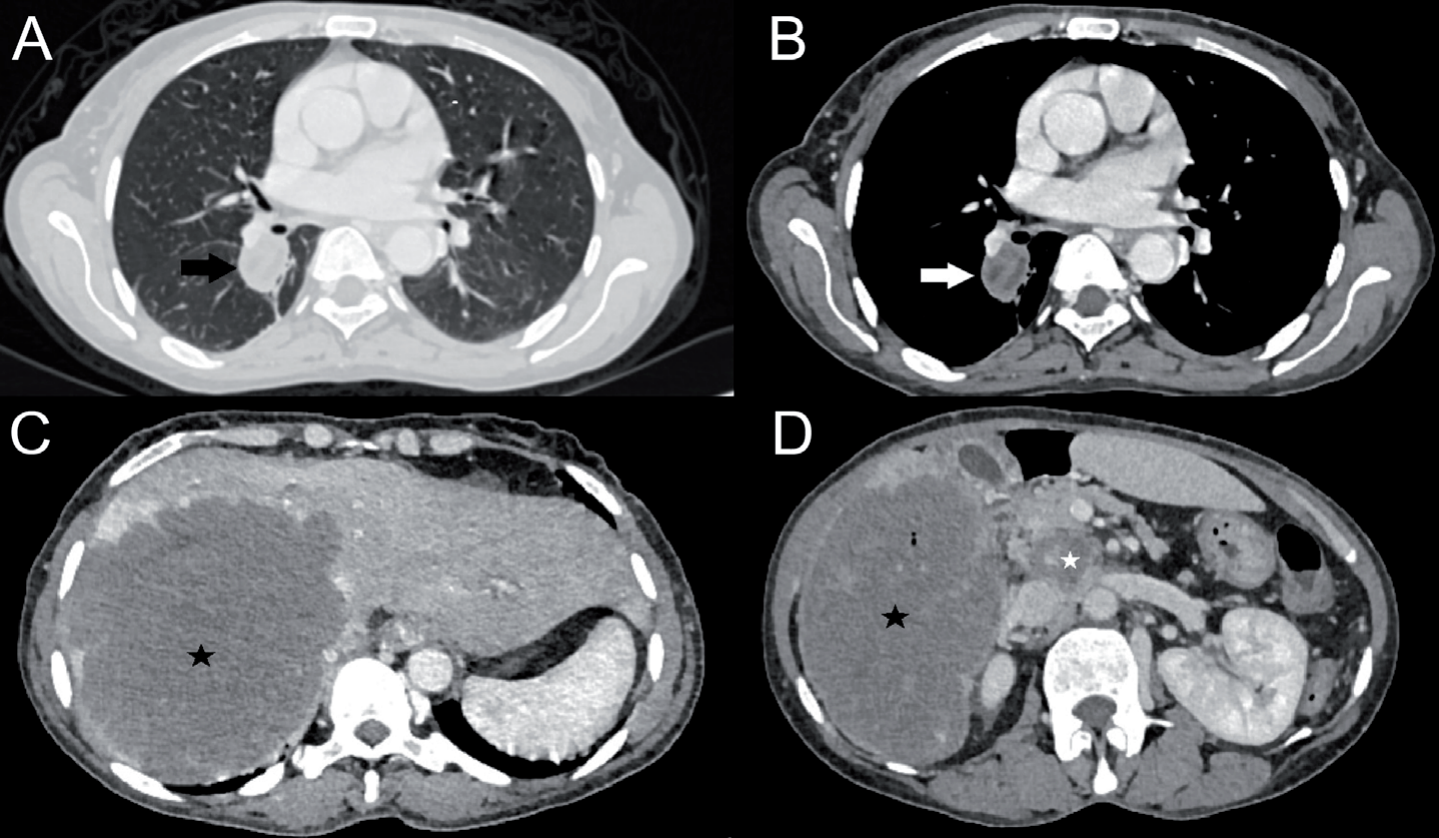
Axial contrast-enhanced computed tomography (CT) images at the level of the lung (A and B) and liver (C and D) show a large alveolar echinococcosis lesion located in the right lobe of the liver (black stars in C and D). Alveolar echinococcosis metastases in the right lung are shown in the same patient (black and white arrows in A and B). In addition, alveolar echinococcosis involvement is observed in the uncinate process of the pancreas (white star in D).

Computed tomography provides more detailed information about the internal nature of the lesion and in terms of invasion. Alveolar echinococcosis appears as a large irregular heterogeneous hypodense mass lesion on computed tomography (Figure 2).^35^ After contrast agent administration, alveolar echinococcosis lesions do not show contrast enhancement. Computed tomography provides a distinct advantage over other radiological methods in showing calcifications within alveolar echinococcosis lesions. Detection of calcifications within the lesions is important in differentiating alveolar echinococcosis from some tumoral lesions. Computed tomography can also detect the relationship of the lesions observed in the liver with the vascular structures, their extrahepatic extension, local invasion to neighboring tissues, and distant organ metastases if any (Figure 3). Because it detects the extent of invasion of the lesions and distant organ metastases, computed tomography is an important guiding method used to decide on the surgical treatment option.^36^

Magnetic resonance imaging features in patients with alveolar echinococcosis typically include heterogeneous signal intensity on T2-weighted images and low signal intensity on T1-weighted images. On T2-weighted images, cystic components show high signal characteristics, while fibrotic areas have low signal characteristics.^37^ Magnetic resonance imaging is the best method to identify cystic components in patients with alveolar echinococcosis.^38,39^ As is known, MRI provides a distinct advantage over computed tomography in detecting disorders in soft tissues. Therefore, MRI is very valuable in evaluating the invasion of surrounding tissues such as the diaphragm, inferior vena cava, and pericardium.^40^ In addition, MR cholangiography has an important role in evaluating biliary system involvement and diagnosing complications related to the biliary system.^41,42^

### Clinical Manifestation and Distant Organ Involvement

**Figure 4. A-C.**
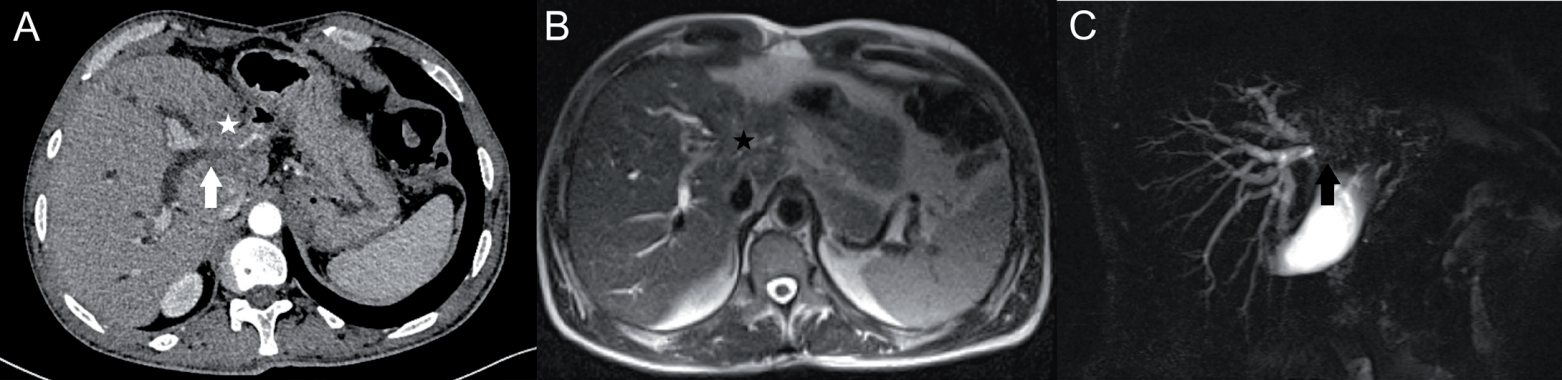
Axial contrast-enhanced computed tomography (CT) image (A), axial T2-weighted magnetic resonance image (B). and thick slap MRCP (Magnetic resonance cholangiopancreatography) image (C) show an alveolar echinococcosis lesion with Klatskin tumor-like involvement is observed in the left lobe of the liver (white and black stars in A and B). Note that the intrahepatic biliary tract is interrupted at the bifurcation level (white and black arrows in A and C), and capsular retraction is observed in the left lobe of the liver adjacent to the lesion.

**Figure 5. A-D.**
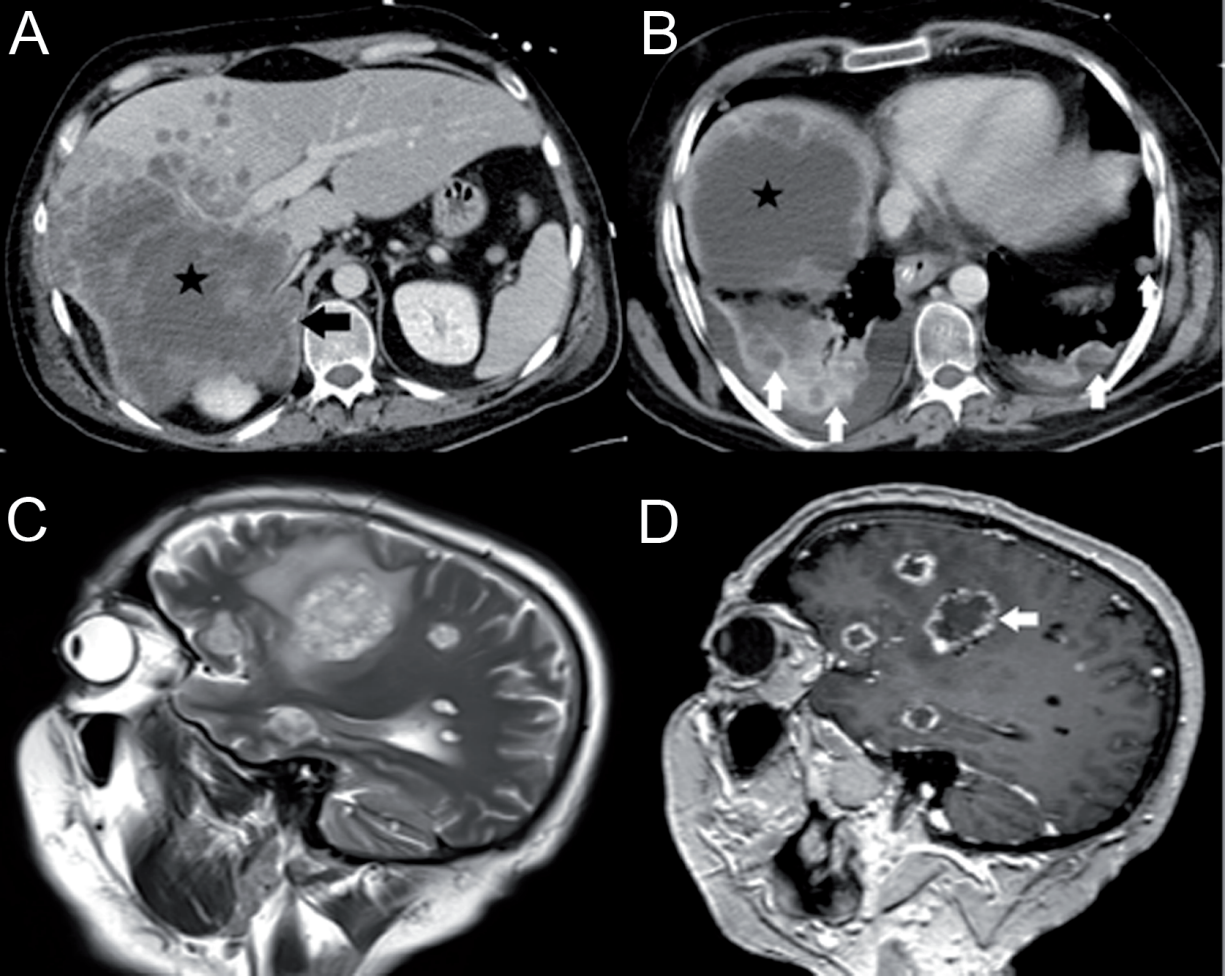
Axial contrast-enhanced computed tomography (CT) imaged at the level of the liver (A) and lung basis (B) shows alveolar echinococcosis lesion in the liver (black stars in A and B) and lung (white arrows in B). In addition to the liver lesion, sagittal T2-weighted magnetic resonance (MR) image (C) and sagittal T1-weighted contrast-enhanced image (D) show multiple alveolar echinococcosis metastases in the brain. The lesion in the liver appears to invade the right adrenal gland with local invasion (black arrow in A). Note that metastases observed in the brain show peripheral contrast enhancement (white arrow in D) and vasogenic edema effect is observed around the lesions on sagittal T2-weighted MR images.

In the early stage, alveolar echinococcosis shows an asymptomatic incubation period that can last from 5 to 15 years. In almost all cases, the disease primarily involves the liver, where it shows tumor-like growth (Figure 4). The larvae may spread by direct invasion into the diaphragm, lung, pancreas, adrenal glands, as well as abdominal lymph node regions and pararenal spaces.^43^ Moreover, as a result of the spread of the larvae via blood and lymphatic vessels, organ involvement such as brain, lung, bone, and breast may occur (Figure 5).^44^

Considering the low incidence of alveolar echinococcosis, distant organ metastases have also been reported extremely rarely.^45^ Cases reported in the literature on extrahepatic involvement of alveolar echinococcosis are often in the form of local invasion of the lesion in the liver. Related to this, involvement of the portal venous system, inferior vena cava, diaphragm, abdominal wall, and lung has been reported more frequently. The lung is the most common organ after the liver in patients with alveolar echinococcosis, and lung involvement may accompany liver lesions in patients.^46^ In lung involvement that can be both via local invasion and via blood spread, cysts may appear as multiple or single nodules and may be unilateral or bilateral.^47,48^ The presence of multiple lesions in the lung may suggest primary lung cancers, multiple abscesses of other infectious agents, or lung metastasis of other malignancies in the differential diagnosis.^49,50^ In addition, miliary pulmonary metastases of alveolar echinococcosis have also been reported.^51^

Distant organ metastasis through blood was reported more frequently as lung and brain metastases. In brain involvement, vasogenic edema is usually found around cystic lesions with multilobular irregular contours.^52^ The cysts are usually surrounded by contrast-enhancing peripheral reactive inflammatory tissue.^53^ Brain lesions can sometimes be difficult to distinguish from brain metastases of malignant diseases. Magnetic resonance imaging has an important role in the identification of brain lesions.^54,55^

However, there is relatively little published literature on some distant organ metastases of alveolar echinococcosis such as skin, vertebrae, heart, ribs, breast, and adrenal glands.^56-58^

## Treatment

The World Health Organization Informal Working Group on Echinococcosis has defined a PNM (P = parasitic mass in the liver, N = involvement of neighbouring organs, and M = metastasis) classification system based on imaging findings to standardize diagnosis and treatment for hepatic alveolar echinococcosis.^59,60^ In all cases suitable for surgical procedures, the first treatment option is surgery.^61^ In addition, long-term medical therapy should be combined with this treatment.^62,63^ Liver transplantation may be considered in selected patients with portal hypertension, ascites, and symptomatic secondary biliary cirrhosis.^64^ In the presence of local spread and distant organ metastases, a multidisciplinary surgical approach is required.^65^ Long-term benzimidazole therapy is mandatory in all patients who are unsuitable for surgery and in patients undergoing radical surgery.^66,67^ Albendazole is a parasitostatic agent and does not usually eliminate *E. multilocularis*.^68,69^ For this reason, patients who are not considered suitable for surgery require lifelong treatment to prevent the growth of the parasite.^70^

Interventional procedures can be used as a treatment option in the treatment of complications of alveolar echinococcosis.^71^ Percutaneous drainage can be applied in lesions with large cystic contents or in lesions with superposed abscess formation. In addition, ERCP (Endoscopic retrograde cholangiopancreatography) can be performed in patients with biliary tract obstruction.^72^

## Conclusion

In conclusion, alveolar echinococcosis is a life-threatening chronic zoonotic disease. Since it is difficult to diagnose the disease at an early stage, it becomes difficult to treat it. Treatment usually requires a multidisciplinary approach. Mass migrations in recent years have led to the emergence of the disease in different geographies. Therefore, in this article, we wanted to review the diagnosis and treatment of alveolar echinococcosis and emphasize the importance of keeping it in mind, especially in cystic lesions of the liver, and the importance of early diagnosis of the disease.
